# Cost-effectiveness of the recommended medical intervention for the treatment of dysmenorrhea and endometriosis in Japan

**DOI:** 10.1186/s12962-018-0097-8

**Published:** 2018-04-10

**Authors:** Ichiro Arakawa, Mikio Momoeda, Yutaka Osuga, Ikuko Ota, Kaori Koga

**Affiliations:** 1 Faculty of Pharmaceutical Sciences, Teikyo Heisei University, Tokyo, Japan; 2 Department of Obstetrics and Genecology, St. Luke International Hospital, Tokyo, Japan; 3 Department of Obstetrics and Genecology, The University of Tokyo, Tokyo, Japan; 4 Department of Genecology, Kurashiki Heisei Hospital, Kurashiki, Japan; 5 Japan Enlightenment Committee in Endometriosis (JECIE), Tokyo, Japan

**Keywords:** Dysmenorrhea, Endometriosis, Self-care, Guideline-based intervention, Cost-effectiveness

## Abstract

**Background and objective:**

This study aims to assess the cost-effectiveness of early physician consultation and guideline-based intervention to prevent endometriosis and/or disease progression using oral contraceptive (OC) and progestin compared to follow-up of self-care for dysmenorrhea in Japan.

**Methods:**

A yearly-transmitted Markov model of five major health states with four sub-medical states was constructed. Transition probabilities among health and medical states were derived from Japanese epidemiological patient surveys and converted to appropriate parameters for inputting into the model. The dysmenorrhea and endometriosis-associated direct costs included inpatient, outpatient visit, surgery, and medication (OC agents, over-the-counter drugs), etc. The utility measure for patients with phase I–IV endometriosis comprised a visual analogue scale. We estimated the cost per quality-adjusted life year (QALY) at a time horizon of 23 years. An annual discount rate at 3% for both cost and outcome was considered.

**Results:**

The base case outcomes indicated that the intervention would be more cost-effective than self-care, as the incremental cost-effectiveness ratio (ICER) yielded 115,000 JPY per QALY gained from the healthcare payers’ perspective and the societal monetary value (SMV) was approximately positive 3,130,000 JPY, favoring the intervention in the cost–benefit estimate. A tornado diagram depicting the stochastic sensitivity analysis of the ICER and SMV from both the healthcare payers’ and societal perspectives confirmed the robustness of the base case. A probabilistic analysis resulting from 10,000-time Monte Carlo simulations demonstrated efficiency at willingness-to-pay thresholds in more than 90% of the iterations.

**Conclusions:**

The present analysis demonstrated that early physician consultation and guideline-based intervention would be more cost-effective than self-care in preventing endometriosis and/or disease progression for patients with dysmenorrhea in Japan.

## Background

Endometriosis is an estrogen-dependent disease with accompanying pain (such as menstrual and chronic pelvic pain), which occurs in young to midlife adult women and accomplish with dysmenorrhea. Women’s life stages are closely related to the onset of endometriosis; the incidence of endometriosis rises in the late 20 s and peaks at approximately 30 years, whereas the most susceptible age for developing adenomyosis is in the late 30 s [1]. In patient with endometriosis, chronic pelvic pain and infertility can lead to deterioration in the quality of life (QOL). In a nationwide survey conducted in 1997, 2.6 million women in Japan of reproductive age had endometriosis [1].

Primary dysmenorrhea is a menstrual disorder defined in the absence of other diseases, such as endometriosis. The initial presentation of primary dysmenorrhea typically occurs in adolescence, and can cause absenteeism and reduced QOL. Dysmenorrhea also adversely affects daily activities including reduced sleep (< 6 h per day) and fewer sport activities [2]. A recent study conservatively estimated the prevalence of moderate-to-severe dysmenorrhea in Japan to be approximately 46.8% among high school students, and those with major symptoms are often underdiagnosed and undertreated [2].

Before the use of gonadotropin-releasing hormone (GnRH) agonists in the pharmacological treatment of dysmenorrhea with endometriosis, acupuncture, self-medication, and Chinese medicines were mainly used for the relief of pelvic pain. However, since 2008, oral contraceptives (OCs) and progestin (dienogest) have been used in the treatment of functional and organic dysmenorrhea in Japan, as they allow for long-term pain control. The current clinical practice guidelines of the Japan Society of Obstetrics and Gynecology (2014 edition) recommend using OCs and dienogest for the treatment of dysmenorrhea with endometriosis [3].

Approximately 1.6 million women in Japan have functional dysmenorrhea, and most do not receive the aforementioned guideline-based treatment in a medical institution during the early stage of the disease [4]. Rather, many women in Japan prefer to deal with their symptoms via self-care (nonvisit) with non-steroidal anti-inflammatory analgesics (NSAIDs) [4], or by consulting with osteopathic clinics. As a result, symptoms deteriorate in most cases, leading to the comorbidity of endometriosis [5]. Tanaka et al. [6] reported that women were experiencing serious menstrual problems to consult a gynecologist as this can improve their QOL in Japan, whereas no such changes were found for patients practicing self-medication with over-the-counter drugs (OTCs). The pain associated with endometriosis causes marked deterioration in the QOL and leads to a serious socioeconomic impact (approximately 380 billion Japanese Yen [JPY] per year) [7]. However, patients and insurers lack full awareness of the guideline-based treatment for dysmenorrhea, and thus far, no reports exist regarding the potential reduction in the socioeconomic impact through early medical intervention.

Therefore, the present study aimed to assess the cost-effectiveness of early physician consultation and guideline-based intervention (simply referred to as the intervention in the remainder of the report) for dysmenorrhea, compared to that for self-care, with a subsequent goal of increasing awareness of the importance and economic impact of the intervention among Japanese political decision-makers and women.

## Methods

### Target population

The target population consisted of patients self-administrating OTCs and/or receiving acupuncture for the treatment of dysmenorrhea.

### Model building and calibration

**Fig. 1:**
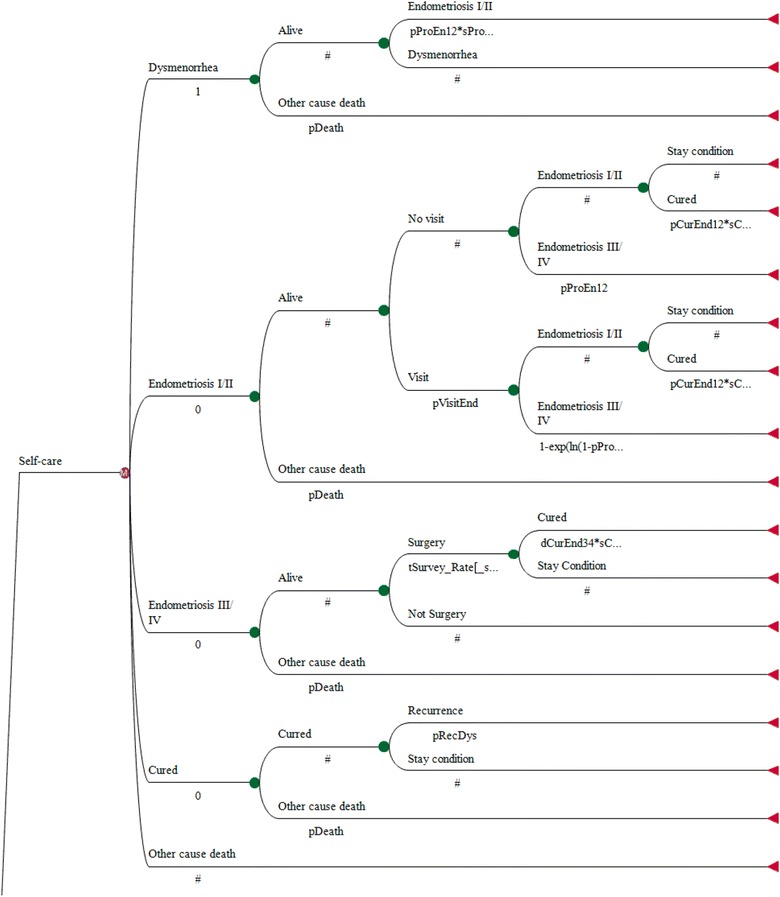
The Markov model used to assess the cost-effectiveness of early medical intervention for dysmenorrhea and endometriosis. A simple Markov model with yearly transmission of five health states (dysmenorrhea, phase I/II endometriosis, phase III/IV endometriosis, cured, and other-cause death) with four sub-medical states (consultation, surgery, recurrence, and stay condition) was constructed based on standard therapeutic and empirical pathways with consensus from gynecologists

No appropriate head-to-head trials evaluating clinical efficacy and economic impact on dysmenorrhea and endometriosis across the various therapies available in Japan yet exist. Therefore, rather than conducting a realistic assessment among therapies, model simulation was chosen, taking advantage of indirect comparisons across therapies. To take into consideration the natural history of disease [8], a simple Markov model with yearly transmission of five health states (dysmenorrhea, phase I/II endometriosis, phase III/IV endometriosis (diagnosed by R-ASRM classification), cured, and other-cause death) and four sub-medical states (consultation, surgery, recurrence, and stay condition) was constructed, based on standard therapeutic and empirical pathways (determined via consensus among gynecologists) (Fig. 1). Transition probabilities among these health and medical states were derived from the following (Table 1a):

**Table 1:** Parameters incorporated into the Markov model

Parameters/utility measures	Valuables	Distribution	Source
*(a) Probabilistic parameters and utility measures*			
Annual incidence of Dysmenorrhea	0.0008–0.007	Age-dependent	National Patient Survey (2011) [9]
Progress from dysmenorrhea to endometriosis I/II	18.4%	Beta	Taketani et al. (1997) [1]
Natural healing in dysmenorrhea	80%	–	Assumption:adjusted by calibration
Natural healing in endometriosis I/II	40%	–	Assumption:adjusted by calibration
Natural healing in endometriosis III/IV	80%	–	Assumption:adjusted by calibration
Recurrence	22.2%	Beta	Taketani et al. (1997) [1]
Visit proportion for dysmenorrhea	0.03–0.5	Age-dependent	Calculation from Taketani et al. (1997) [1] and National Patient Survey (2011) [9]
Visit proportion for endometriosis	0.0124–0.0307	Age-dependent	
Surgery for endometriosis	6.0%	Normal	Tanaka et al. (2013) [6]
Proportion of OTC use for self-medication	87.1%	Normal	Tsutsumi et al. (2002) [28]
Annual other-cause death	Age-dependent	–	Life-time table (2013) [10]
Odds ratio for risk reduction in the development of endometriosis I/II	0.40	LogNor	Vessey et al. (1993) [11]
Odds ratio for risk reduction in the progression to endometriosis III/IV	0.10	LogNor	Tutunaru et al. (2006) [12]/Duffy et al. (2014) [13]
Utility for dysmenorrhea	0.637	LogNor	Assumed that utility for dysmenorrhea is the same as that for endometriosis I/II
Utility for endometriosis I/II	0.637	LogNor	Institution-based QOL survey using visual analogue scales
Utility for endometriosis III/IV	0.549	LogNor	
Utility for cured	1.000	–	Assumption

Costs (JPY)/utilizations	Valuables	Distribution	Source
*(b) Cost parameters and resource utilizations*			
Annual frequency of visits for dysmenorrhea	4	–	Assumption
Visit cost for dysmenorrhea	7529	LogNor	Resource utilization survey (2011) [16]
Annual frequency of visits for endometriosis	4	–	Assumption
Visit cost for endometriosis	11,291	LogNor	Resource utilization survey (2011) [16]
Annual inpatient cost (excl. surgery cost)	207,661	LogNor	
Cost of surgery (mild)	288,080	LogNor	National insurance price list established by the surgeons’ group (2014) [29]
Cost of surgery (complex)	456,667	LogNor	
Proportion of complex cases^a^	45.2%	LogNor	Nagata et al. (1982) [30]
Annual OTC cost used (self-care)	19,243	LogNor	Recalculation of raw data from Tanaka et al. (2013) [6]
Annual self-medication cost (self-care)	13,715	LogNor	
Productivity loss (half year, self-care)	184,625	LogNor	
Productivity loss (half year, guideline-based intervention)	39,546	LogNor	

*OTC* over the counter, US$ 1 = approximately 120 JPY

^a^ Complex cases were defined as cases diagnosed as either severe or extensive using the American Fertility Society Classification of Endometriosis (AFS Classification)

1. The National Patient Survey of 2011 [9], which was used to estimate the annual age-dependent incidence of dysmenorrhea in adolescent and adult women, with consideration of the life-time [10];
2. The Japan Burden of Illness (BOI) survey [6]; and
3. Taketani et al.’s Health Sciences Research (National Grant-in-Aid) report [1], which was used to estimate the recurrence rates for dysmenorrhea and endometriosis.

The probabilities were converted to single and/or age-dependent parameters for model input. The single transition probabilities among these health states were converted into parameters for the model by assuming that the probabilities followed a non-parametric and exponential survival distribution (see Eq. 1).

1 $$p = 1 - e^{ - rt}$$

where *p* is the probability, *r* is the hazard ratio (HR), and *t* is the time (years).

To generate the odds ratios (ORs) for risk reduction in the accompaniment of endometriosis phase I/II and the deterioration of phase II to phase III/IV, we systematically conducted a literature review (PICO statement: women with endometriosis [if possible, Japanese women], medical or surgical intervention, pain-free rate at 12 months, placebo or diagnostic laparoscopy; literature search databases: PubMed and the Cochrane library) for reports on self-care for endometriosis phases II to III/IV with an evaluation duration of over 12 months. Vessey et al. [11] reported that the relative risk (RR) for OCs in the 12-month rate of endometriosis was 0.4 (95% confidence interval [CI] 0.2–0.7), which was used as an estimate for risk reduction in the accompaniment of endometriosis phase I/II. In addition, Tutunaru et al. [12, 13] reported that laparoscopic ablation or excision was associated with decreased overall pain at 12 months compared to that with diagnostic laparoscopy only (OR 10.00, 95% CI 3.21–31.17, p = 0.001), respectively. From this, we converted the reciprocal number of the aforementioned OR to an OR of 0.10 for endometriosis phases II to III/IV.

To estimate risk reduction in the accompaniment of endometriosis phase I/II and deterioration of phase II to phase III/IV, we estimated new model parameters using the following equation (see Eq. 2). The exponential distribution, as in Eq. 1, was characterized by a constant HR, independent of time, including age. A high *r* value indicated a high risk and short survival; a low *r* value indicated low risk and long survival [14].

2 $$r_{RR} = {\text{ r}}_{d} \times {\text{ OR}}$$

Where *r*_*RR*_ represents the HR after risk reduction, r_*d*_ represents the HR before risk reduction, and OR is derived from Vessey et al. [11], Tutunaru et al. [12, 13]. Finally, HRs after risk reduction were converted using Eq. 1. To calibrate and validate the model, the simulated prevalence of endometriosis generated by the model was externally compared with that for reported cumulative [15] and age-specific prevalences [9].

### Cost variables and cost components in different perspectives

The direct costs associated with dysmenorrhea and endometriosis, including inpatient care, outpatient visits, surgery, and medication costs (including OCs and OTCs) (Table 1b) were determined for both the healthcare payers’ and societal perspectives. The average expense for inpatient and outpatient visits (inclusive of medical drug cost) was calculated using the Resource Utilization Survey of 2011 [16], which was conducted widely across Japan and is considered highly representative, and the Patient Survey of 2011 [9]. Drug costs associated with OTCs were derived from the Japan BOI survey [6]. Opportunity costs due to productivity losses associated with dysmenorrhea and endometriosis were separately derived from the recalculation of raw data based on the BOI survey [6].

In the cost-effectiveness analysis from the perspective of the healthcare payer, only the full direct medical costs paid by national health insurance were considered. In the cost–benefit analysis from the societal perspective, full direct medical costs, non-medical direct costs, and opportunity costs were considered.

### Health outcomes

The primary health outcome was the quality-adjusted life-year (QALY) from the perspective of the healthcare payers. As literature searches failed to provide evidence on utility measures for Japanese women with disease, the utility measures for endometriosis comprised a Visual Analogue Scale (VAS), with ratings from death (0.000) to perfect health (1.000) (Table 1a), used in an institution-based and cross-sectional survey carried out at the University of Tokyo and Kurashiki Heisei Hospital (the data analysis was implemented at Teikyo Heisei University). The investigators received ethical approval for the QOL survey at the involved medical institutes (Approval No.: 10556 [The University of Tokyo], H26-003 [Kurashiki Heisei Hospital], 26-013 [Teikyo Heisei University]), and informed consent was provided by all eligible subjects. The prophylactic effectiveness of treatment on the incidence of endometriosis was defined as a secondary health outcome.

### Cost-effectiveness and cost–benefit assessment

The cost-effectiveness of the intervention relative to that for self-care was assessed using the incremental cost-effectiveness ratio (ICER) at a time horizon of 23 years; we assumed that a single cohort of girls aged 12 years (the mean age of first menstruation) were examined longitudinally to an age of 35 years. An annual discount of 3% for both the cost and outcome was considered, in accordance with the guidelines on economic evaluation established by the International Society for Pharmacoeconomics and Outcomes Research [17]. If the ICER was less than the willingness to pay (WTP) threshold of five million JPY (equivalent to US$ 42,000, assuming an exchange rate of 1 US$ to 120 JPY) per QALY gained [18], the intervention was assessed for cost-effectiveness from the healthcare payers’ perspective, with excluded non-medical (OTC usage and acupuncture) and patient-time opportunity costs.

In the cost–benefit analysis from the societal perspective, full direct and opportunity costs were considered in the cost component. The societal monetary value (SMV) was calculated using the following equation (Eq. 3), and as an SMV equal to zero reflects the break-even point, a positive value favors the intervention.

3 $${\text{SMV }} = {\text{opportunity cost saved }}{-}{\text{ full direct cost consumed}}$$

### Sensitivity analyses

Uncertainty was taken into consideration in the simulated cost-effectiveness (ICER) and cost–benefit (SMV) analyses from both perspectives (healthcare payers and societal) via stochastic uncertainty analysis using a Tornado diagram analysis. The Tornado diagram depicting the stochastic sensitivity analysis of the ICER and SMV was drawn to reveal parameters influencing the base case, and to demonstrate the robustness of the base case.

In addition, a Bayesian approach to the probabilistic analysis of the ICER with 10,000-time Monte Carlo (MC) simulations was performed to identify an efficient strategy for the WTP variable thresholds, as indicated by the cost per QALY gained and the obtained 95% CIs for the incremental cost and effectiveness with p value. The cumulative probabilities (*p*_*(0*<*X*<*5M)*_) for the medical intervention and self-care were illustrated in cost-effectiveness acceptability curves. The chosen distribution for each of the parameters is indicated in Table 1a and b.

In general, continuous variables with an interval scale were assumed to follow a normal distribution with mean and standard deviation (SD) (*N* [*μ*, *σ*^*2*^]), while continuous variables with a ratio scale were assumed to follow a lognormal distribution with logarithmic mean and SD (*LN* [*μ, σ*^*2*^]). Variables with a binomial scale such as disease-progression and recurrence rates were assumed to have a normal sampling distribution in the generation of the CIs. In addition, some variables with a binomial distribution such as “disease-progression” and “recurrence” were assumed to follow a beta distribution (*B* [*α, β*]). Finally, the OR for risk reduction in the accompaniment of endometriosis with the intervention was assumed to follow a lognormal distribution with logarithmic mean and SD (*LN* [*μ*, *σ*^*2*^]) [19].

### Analytical software

Analyses were performed using Windows Excel 2013 (Microsoft, Inc., Washington, US) and TreeAge PRO Healthcare 2015 (TreeAge Software, Inc., Massachusetts, US).

## Results

### Model calibration and external validity

**Fig. 2:**
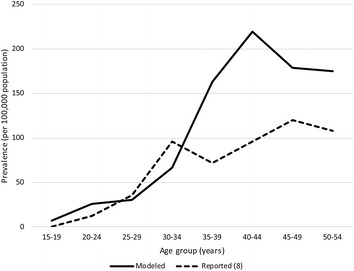
Model calibration and validation through comparison with realistic statistics. The simulation demonstrated the validity of the model in computing prevalence of approximately 210,000 cases with endometriosis, similar to that reported in a national survey (approx. 247,000^a^; Terakawa et al. [15]); however, a discrepancy in prevalence for age-groups of 35 years or more existed. ^a^number of patients with endometriosis plus adenomyosis

The survival trends for the prevalence of dysmenorrhea, compared to those from the National Patient Survey [8], are illustrated in Fig. 2. The simulation demonstrated the validity of the model, with a computed cumulative prevalence of approximately 210,000 cases of endometriosis, similar to those retrieved from Terakawa et al. [15]; however, discrepancies existed in the prevalence for age groups of 35 years or more.

### Health outcome evaluation for the modeled prevalence of endometriosis

The simulation demonstrated that the accompaniment of endometriosis was reduced by 95% when patients with dysmenorrhea received the intervention compared to that for self-care.

### Cost-effectiveness and cost–benefit for a base case from differential perspectives

From the healthcare payers’ perspective, the ICER was approximately 115,000 JPY ($958) per QALY gained, with gains in the incremental cost of 300,000 JPY (US$ 2500) and gains in the incremental effectiveness of 2.6 QALYs, retrospectively (Table 2). In the cost–benefit estimate, the SMV was approximately positive 3,130,000 JPY (US$ 26,000).

**Table 2:** *Cost-effectiveness and -benefit for the base case*

Group	Expected cost (JPY)	Expected effectiveness (QALYs)
(a) The base case from the perspective of healthcare payers		
Guideline-based intervention^a^	326,806	14.9
Self-care^a^	27,758	12.3
Incremental	299,048	2.6

Group	Total cost^b^ (JPY)	
(b) The base case from the societal perspective		
Guideline-based intervention	1,643,076	
Self-care^b^	4,768,899	
Societal monetary value (SMV)	Δ3,125,823	

The incremental cost-effectiveness ratio (ICER) was approximately 115 thousand JPY ($958) per quality-adjusted life-year (QALY) gained, retrospectively. Thus, the model simulation revealed that early physician consultation and guideline-based intervention would be more cost-effective than self-care, as the aforementioned ICER is below the willingness to pay (WTP) threshold

In the cost–benefit analysis, the aforementioned SMV favored early physician consultation and guideline-based intervention

^a^ Medical direct costs, which were established from the National Health Insurance scheme, consist of outpatient visits (inclusive of drugs), inpatient care, and surgery

^b^ Total costs consist of medical direct, non-medical and opportunity costs

### Stochastic and probabilistic sensitivity analyses from the differential perspective

**Fig. 3:**
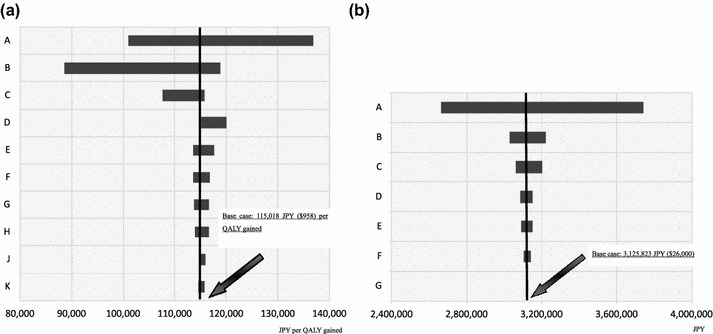
Tornado diagram **a** The incremental cost-effectiveness ratio (ICER) Tornado diagram used to assess the robustness of the base case analysis from the perspective of healthcare payers. A Tornado diagram depicting the results of the stochastic sensitivity analysis for ICER revealed that the cure rate for dysmenorrhea resulting from the guideline-based intervention influenced the base case; however, the robustness of the base case was confirmed. Indices of parameters (**a**) A: Cure rate for dysmenorrhea (0.643 to 0.957). B: Odds ratio for the development of dysmenorrhea (0.2 to 0.7). C: Utility for endometriosis III/IV (0.15* to 0.557). D: Proportion of visits in patients with endometriosis (0.0 to 0.07). E: Progression to endometriosis I/II (0.179 to 0.189). F: Discount rate (0.01 to 0.05). G: Utility for dysmenorrhea (0.63 to 0.644). H: Recurrence of dysmenorrhea (0.206 to 0.239). J: Cure rate of endometriosis I/II (0.322 to 0.478). K: Utility for endometriosis I/II (0.63 to 0.644). *: To take into account worst case scenario, lower value of the utility measuring for endometriosis III/IV was derived from the external criteria [20]. **b** The incremental cost Tornado diagram used to assess the robustness of the base case analysis from the societal perspective. A Tornado diagram depicting the results of the stochastic sensitivity analysis for IC revealed that the discount rate resulting from the guideline-based intervention influenced the base case; however, the robustness of the base case was confirmed. Indices of parameters (**b**) A: Discount rate (0.01 to 0.05). B: Recurrence of dysmenorrhea (0.206 to 0.239). C: Cure rate of endometriosis I/II (0.322 to 0.478). D: Odds ratio for the development of dysmenorrhea (0.2 to 0.7). E: Progression to endometriosis I/II (0.179 to 0.189). F: Cure rate for dysmenorrhea (0.643 to 0.957). G: Proportion of visits in patients with endometriosis (0.0 to 0.07)

Although a Tornado diagram depicting the results of the stochastic sensitivity analysis for ICER from the healthcare payer’s perspective showed that the cure rate for dysmenorrhea resulting from the intervention influences the base case, the robustness of the base case was confirmed (Fig. 3a). In addition, although the Tornado diagram on the SMV from the societal perspective showed that the discount rate influences the base case, the robustness of the base case was confirmed (Fig. 3b).

**Fig. 4:**
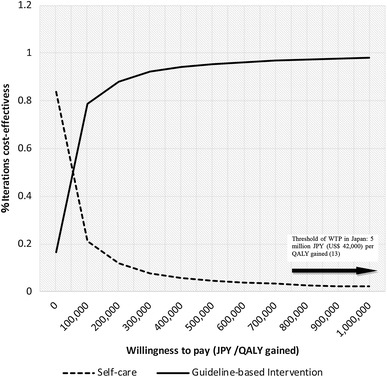
Cost-effectiveness acceptability curve for early medical intervention in dysmenorrhea and endometriosis from the perspective of healthcare payers. The results of a probabilistic analysis with 10,000-time Monte Carlo simulations are illustrated by cost-effectiveness acceptability curves and demonstrated the efficacy of medical intervention at a willingness to pay threshold of less than a million JPY per quality-adjusted life-year gained in more than 90% of the population

The results from a probabilistic analysis with 10,000-time MC simulations demonstrated efficiency at a WTP threshold of 100,000 JPY ($884) per QALY gained and five million JPY (US$ 42,000) per QALY gained [18] in 80% and more than 90% of the iterations, respectively, as illustrated in cost-effectiveness acceptability curves (Fig. 4). The MC simulations on incremental cost and effectiveness, with 95% CIs, yielded 320,282 JPY (95% CI 277,383, 363,181, p < 0.001) in cost and 2.57 QALYs gained (95% CI 2.55, 2.59, p < 0.001) in effectiveness, where normal distributions in cost and effectiveness were assumed.

## Discussion

We aimed to assess the economic impact of early physician consultation and guideline-based intervention for dysmenorrhea compared to that for self-care from two perspectives (the healthcare payers’ and societal perspectives) in Japan. In summary, from both perspectives, the intervention would be more cost-effective than self-care. The stochastic sensitivity analyses for the ICER and SMV confirmed the robustness of the base case. Moreover, a probabilistic analysis demonstrated efficiency at a WTP threshold of 115,000 JPY ($958) per QALY gained and five million JPY (US$ 42,000) per QALY gained [18] in 80% and more than 90% of the iterations from the healthcare payers’ perspective.

To assess the cost-effectiveness of early physician consultation and guideline-based intervention compared to that for self-care in Japan, a simple Markov model with yearly transmission of five health states and four sub-medical states was constructed (Fig. 1). Sanghera et al. [20] reported on the pharmaceutical treatment of endometriosis recurrence following surgery from the perspective of the United Kingdom’s National Health System using a model-based economic evaluation. As the analysis provided a post-surgical assessment of the 36-month costs and outcomes for endometriosis among alternatives, it was modeled as a state transition after surgery. Therefore, face validity differs between the present model and that used in Sanghera et al. [20].

In European and other Asian countries, numerous studies have been conducted examining the cost-effectiveness and clinical efficacy of acupuncture treatment for dysmenorrhea compared to that for conventional treatment and the waiting approach [21, 22]. In addition, the use of GnRH agonists for 3 months has been reported to be a cost-effective approach in the primary diagnosis and treatment of endometriosis in women with pelvic pain [23]. However, the cost-effectiveness of NSAIDs for dysmenorrhea and endometriosis has not yet been fully assessed. Moreover, the current clinical practice guidelines for Japan [3] recommend the use of OCs in the treatment of dysmenorrhea. Clinical trials (randomized double-blind controlled trials) conducted in Japan have demonstrated that a 4-week treatment with OCs led to statistically significant improvements in clinical endpoints (over pre-treatment assessments) in patients with functional and organic dysmenorrhea [24]. Thus, in Japan, as well as in European and other Asian countries, a need exists for the evaluation of the cost-effectiveness of the currently recommended medical intervention using established clinical evaluation methods.

Prior to conducting the cost-effectiveness analysis, the model was calibrated by comparing the cumulative and age-specific prevalences reported in existing cohort surveys [9, 15] with the simulated prevalences generated by the model. The simulated prevalences for women aged 15–19 and 30–34 years, as well as the simulated cumulative prevalence, were similar to those found in the aforementioned cohort surveys. However, the simulated prevalences for women aged 35 years or more tended to be much higher compared to that previously reported, possibly because the present study assumed a recurrence of dysmenorrhea after remission due to medical intervention. Alternatively, we should have taken into consideration the remission of endometriosis due to childbirth, as this may contribute to a decreased incidence of disease in midlife women. Thus, we attempted to search for a proper parameter value to represent the remission of endometriosis due to childbirth; however, we found that no such valid parameter is available at this moment. Even if future incurring costs were converted to current values by a discounting of 3% in the analysis, there would be less impact on the overall cost. Therefore, we decided to proceed with the cost-effectiveness analysis, without consideration of the remission of endometriosis due to childbirth.

The proportion of cases progressing to or complicated with endometriosis was estimated for early physician consultation and guideline-based intervention as well as for self-care. The resulting estimations suggested that the proportion of such cases decreased by approximately 95% for the intervention compared to that for self-care. A national case–control study reported that women with endometriosis had an approximately eightfold increased risk of dysmenorrhea [25]. Moreover, a qualitative interview-based cohort study conducted in the United Kingdom concluded that an early diagnosis was critical to reduce suffering at physical, emotional, and social levels [26]. Therefore, we conclusively emphasize that early physician consultation and guideline-based intervention in clinical practice should be considered to reduce the complications of endometriosis.

From the perspective of the healthcare payers, the ICER was approximately 115,000 JPY ($958) per QALY gained, with gains in the incremental cost of 300,000 JPY (US$ 25,000), and gains in the incremental effectiveness of 2.6 QALYs; therefore, the intervention is cost-effective, as the ICER is below the threshold of five million JPY (US$ 42,000) per QALY gained [18]. In addition, a stochastic sensitivity analysis on ICER was performed. The OR for progression from a functional to an organic pathophysiology was shown to have a major influence on the analytic results, but no influence on the base case; thus, the robustness of the base case was demonstrated.

Overall, the present results suggest that early physician consultation and guideline-based intervention is more cost-effective than self-care in patients with dysmenorrhea in Japan. The findings provide meaningful information for the future education of patients and governmental authorities regarding the intervention of dysmenorrhea. Most patients with potential functional dysmenorrhea do not receive proper medical intervention in the early stages of the disease (the proportion visiting a gynecologist is less than 20%), and instead perform self-care with NSAIDs, osteopathic therapy, and/or acupuncture [27]. Thus, the disease is often aggravated and progresses to or is complicated by endometriosis, with great socioeconomic impact. Therefore, the present findings are also meaningful in encouraging individual patients to consult medical institutions.

Given the acknowledged shortcomings of the data (e.g., the use of variables from different data sources) and the use of numerous assumptions and adjustments, our conclusions should be considered with care. In addition, no valid parameter estimates could be obtained from Japanese statistical and cohort data to represent the proportions of remission of dysmenorrhea and endometriosis with self-care; therefore, we adjusted the values for these variables by comparing the modeled estimations with those from previous studies. Because these variables were shown in the Tornado diagram to influence the results of the analyses conducted in our study, recalculations will be required with the future acquisition of valid parameter estimations. In addition, although the utility measure for the state of dysmenorrhea was also a meaningful factor that influenced the study results, the state of endometriosis phase I/II was used. A rigorous distinction between functional and organic dysmenorrhea associated with endometriosis relies on a pathological diagnosis based on laparoscopic surgery or laparotomy, and cannot be based on subjective symptoms alone. Therefore, the consideration of the utility value for the state of dysmenorrhea as equivalent to endometriosis I/II is appropriate. In the present study, utilities measured by VAS were incorporated into the model. Drummond et al. [31] stated that preferences for chronic states could be measured on a rating scale method. Separately, we also measured the utility for endometriosis I/II and III/IV using the EQ-5D-3L. The EQ-5D could not, however, detect utility values such as physical, mental, and social disabilities in the aforementioned health states, except for pain/discomfort. Finally, the present study evaluated the health economics for a “guideline-based intervention”, and did not examine each therapeutic agent individually. Thus, a future health economics study of each individual therapeutic agent is required.

## Conclusions

The present analysis demonstrated that an early physician consultation and guideline-based intervention would be more cost-effective than self-care for patients with dysmenorrhea in Japan. The provided information regarding the cost-effectiveness of the intervention can assist in decision-making for patients with dysmenorrhea, as well as health-policy makers.
